# SP-D attenuates LPS-induced formation of human neutrophil extracellular traps (NETs), protecting pulmonary surfactant inactivation by NETs

**DOI:** 10.1038/s42003-019-0662-5

**Published:** 2019-12-16

**Authors:** Raquel Arroyo, Meraj Alam Khan, Mercedes Echaide, Jesús Pérez-Gil, Nades Palaniyar

**Affiliations:** 1 Department of Biochemistry, Faculty of Biology, Complutense University, 28040 Madrid, Spain; 2 Research Institute “Hospital 12 de Octubre (imas12)”, 28041 Madrid, Spain; 3 Program in Translational Medicine, Peter Gilgan Centre for Research and Learning, The Hospital for Sick Children, Toronto, ON M5G 1X8 Canada; 4 Department of Laboratory Medicine and Pathobiology, and Institute of Medical Sciences, Faculty of Medicine, University of Toronto, Toronto, ON Canada

**Keywords:** Neutrophils, Translational research, Immune cell death

## Abstract

An exacerbated amount of neutrophil extracellular traps (NETs) can cause dysfunction of systems during inflammation. However, host proteins and factors that suppress NET formation (NETosis) are not clearly identified. Here we show that an innate immune collectin, pulmonary surfactant protein-D (SP-D), attenuates lipopolysaccharide (LPS)-mediated NETosis in human neutrophils by binding to LPS. SP-D deficiency in mice (*Sftpd*^−/−^) leads to excess NET formation in the lungs during LPS-mediated inflammation. In the absence of SP-D, NETs inhibit the surface-active properties of lung surfactant, essential to prevent the collapse of alveoli, the air breathing structures of the lungs. SP-D reverses NET-mediated inhibition of surfactant and restores the biophysical properties of surfactant. To the best of our knowledge, this study establishes for the first time that (i) SP-D suppresses LPS-mediated NETosis, (ii) NETs inhibit pulmonary surfactant function in the absence of SP-D, and (iii) SP-D can restore NET-mediated inhibition of the surfactant system.

## Abstract (web summary)

Arroyo et al. show that pulmonary surfactant protein-D (SP-D), an innate immune collectin, neutralizes LPS and attenuate LPS-mediated formation of neutrophil extracellular traps (NET) in human neutrophils. This study suggests that SP-D sustains the breathing function of the lung during sepsis by reversing NET-mediated inhibition of lung surfactant.

## Introduction

Neutrophils infiltrate into the alveolar spaces upon sensing microbial insults^1,2^. LPS is a key bacterial component responsible for inducing the formation of neutrophil extracellular traps (NETs), a process generally known as NETosis^3–7^. NETs are unique mesh-like structures made of decondensed chromatin fibers released by neutrophils decorated with cytotoxic granular proteins^3,8^. NETosis may occur via NADPH oxidase 2 (NOX)-dependent and NOX-independent pathways^9,10^. Bacterial cell wall components (e.g., LPS) and phorbol myristate acetate (PMA) induce NOX-mediated production of reactive oxygen species (ROS) and subsequent NETosis^7,11^. Agonists (e.g., A23187, ionomycin) that promote calcium influx and increase mitochondrial ROS production induce NOX-independent NETosis. Increase in intracellular calcium also activates peptidyl arginine deiminase 4 (PAD4) that citrullinates histone 3 (citH3), promoting chromatin decondensation and subsequent faster NETosis^11–15^. Under in vivo conditions, both forms of NETosis could occur simultaneously due to the involvement of multiple activators liberated during inflammation^16^.

Although NETs are beneficial for controlling acute infection, excess or dysregulated NET formation is deleterious to surrounding tissues and associated with lung injury and inflammatory lung diseases^1,17–19^. The function of lung surfactant (LS), which allows compression and expansion of alveoli during normal breathing without collapse^20,21^, could be compromised by the presence of increased amounts of NETs in the airways. This is a potential contributor to respiratory failure in lung pathologies^22^. The hydrophobic surfactant proteins (SP-B and SP-C) and phospholipids in LS are essential for surfactant biophysical function^23^. By contrast, the hydrophilic multifunctional surfactant proteins SP-A and SP-D present in association with lung surfactant play key roles in the innate immune defense and homeostasis of the lungs by interacting with various targets (carbohydrates, DNA, neutrophils, surfactant lipids)^24^. Therefore, hydrophilic surfactant proteins could protect the lungs during inflammation by reducing the negative effect of the NETs on lung surfactant.

In particular, SP-D recognizes various components of microbial pathogens^25^ and participates in lung surfactant homeostasis^26^. SP-D is found as a collection of oligomeric forms^27^. The protein possesses long collagen-like segments and globular heads at the C-terminal end, which are carbohydrate recognition domains (CRD or lectin domains). SP-D binds to LPS of certain bacterial strains through the CRD domain in a calcium-dependent manner^28^. Moreover, SP-D can interact with DNA^29^ and NETs^30^. However, it is unknown whether SP-D has a role in modulating LPS-induced NETosis or whether SP-D could protect LS components from potential NET-mediated inhibition. In this study, we have investigated whether (i) SP-D suppresses NETosis, (ii) NETs inhibit surfactant function in the absence of SP-D, and (iii) SP-D protects LS from NET-mediated inhibition in vitro and in vivo. Answering these questions is important for understanding the fundamental regulatory mechanisms of NETosis in vivo. Furthermore, we have analyzed how NETs affect LS biophysical function, which would be translated in vivo in the ability to breathe with ease, and the key factors that regulate/alleviate these complications. This study shows that SP-D is a key regulator of NETosis and NET-related LS biophysical functions.

## Results

### SP-D binds LPS and suppresses LPS-mediated NETosis

**Fig. 1:**
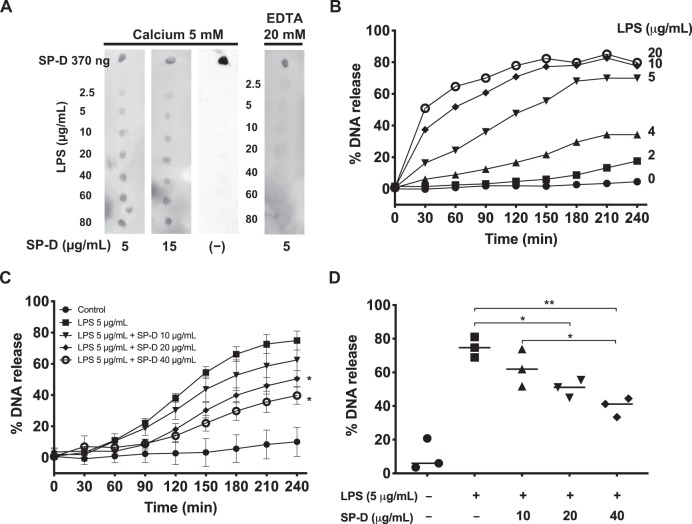
SP-D binds LPS (O128:B12) and suppresses LPS-induced NETosis. **a** Dot blots showing that SP-D binds to LPS. LPS (O128:B12) at different concentrations was dotted onto a membrane, which was incubated with SP-D at different concentrations in the presence of 5 mM CaCl_2_ or 20 mM EDTA. Human SP-D was obtained from the BAL of patients suffering from proteinosis. **b**–**d** NETosis kinetics in neutrophils isolated from healthy human peripheral blood, as assessed by SytoxGreen plate reader assays, where the %DNA released by the cells in the presence of 0.5% (v/v) Triton was considered as 100%. **b** LPS dose-dependently induces NETosis. LPS at concentrations of ≥5 μg/mL induced substantial amount of NETosis. **c** SP-D dose-dependently suppresses LPS (5 μg/mL)-induced NETosis (*n* = 3, the values shown are the average from three individual experiments performed on different days; Two-way ANOVA with Bonferroni’s multiple comparison post-test F(8,90) = 147.7, *p* < 0.0001; post-test *p < 0.05). **d** The differences in NETosis at 240-min time points shown in panel C (*n* = 3, the values shown are the average from three individual experiments performed on different days, One Way ANOVA with Tukey’s multiple comparison post-test, F(1.477,2.953) = 27.98, *p* = 0.0127; *p < 0.05; **p < 0.01). Error bars represent standard deviation. See Supplementary Fig. 4 for positive and negative controls for SytoxGreen Assay.

SP-D binds to LPS of certain strains of bacteria^31–33^. Dot blot analysis was performed to determine the SP-D binding to LPS *Escherichia coli *O182:B12. Different concentrations of LPS (2.5 to 80 μg/mL; in a volume of 2 μL) were placed as dots on a membrane, and incubated with 5 μg/mL or 15 μg/mL of SP-D, in the presence of 5 mM CaCl_2_ or 20 mM EDTA. A negative control experiment was carried out in the same conditions in the absence of SP-D. The amount of SP-D bound to LPS was determined by developing the dot blot after detecting the SP-D with anti-SP-D antibodies. Blot images showed that SP-D binds to LPS O182:B12 in a dose-dependent manner (Fig. 1a). Moreover, calcium was required for this interaction, suggesting that SP-D binds LPS through its CRDs.

Since LPS (O128:B12) strain is different from other strains previously tested elsewhere^6^, we have first determined the ability of this LPS strain to induce NETosis (a wide range of dosage titration) by using a SytoxGreen (cell impermeable DNA binding dye)-based assay with human neutrophils. LPS O182:B12 induces NETosis in a dosage dependent manner (Fig. 1b). Based on these results, 5 μg/mL of LPS was chosen as the optimal working concentration for most of the remaining experiments.

After determining the interaction of SP-D with LPS O128:B12, we investigated whether the presence of SP-D could modulate LPS-induced NETosis. For that, a SytoxGreen assay was conducted as stated above at a 5 μg/mL LPS concentration in the absence or presence of different concentrations of SP-D. Before adding LPS to the cells, the agonist was incubated with SP-D, at a final calcium concentration of 0.5 mM. The data on the percentage of DNA release show that SP-D suppresses LPS-mediated NETosis in a dose-dependent manner (Fig. 1c, d and Supplementary Fig. 2).

Furthermore, we tested the effect of independently adding SP-D and LPS to neutrophils. We first added different concentrations of SP-D (5 or 10 μg/mL) to isolated neutrophils. The SP-D pre-treated neutrophils were then activated by LPS, and NETosis kinetics was assessed for 240 min (Supplementary Fig. 3). This approach would favor the possible interaction of SP-D with the receptors present on the neutrophils. However, LPS-mediated NET formation was not suppressed in these SP-D pre-treated cells. A control sample prepared as in the previous experiments (premixing LPS and SP-D) was also added as a positive control (Supplementary Fig. 3 “PM-LPS + SP-D”), which showed suppression of NETosis. Therefore, LPS-SP-D interaction is required during the LPS-mediated suppression of NETosis.

To confirm the specificity of this effect, we conducted the NETosis assays using other agonists. NETosis induced by other known agonists such as PMA (NOX-dependent NETosis) or ionomycin (NOX-independent NETosis) was not modulated by SP-D. As neutrophils underwent NETosis in these conditions, the percentage of DNA released was not altered by the presence of SP-D (Supplementary Fig. 4). Therefore, SP-D-mediated suppression is specific to LPS-mediated NETosis, but it does not interfere with PMA- or ionomycin-activated neutrophils.

Collectively, these findings show that SP-D binds to LPS (O128:B12) in a dose-dependent manner and as a consequence, SP-D attenuates or suppresses LPS-induced NETosis. Moreover, calcium is required for this function. PMA- or ionomycin-mediated NETosis is not altered by the presence of SP-D.

### Imaging confirms that SP-D suppresses LPS-mediated NETosis

**Fig. 2:**
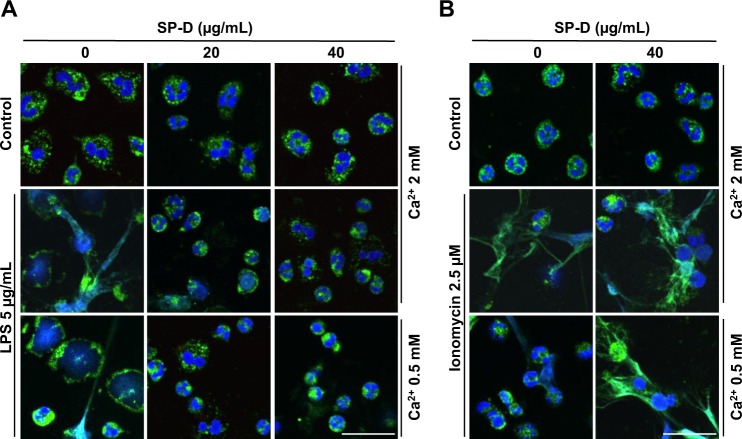
Immunostained confocal microscopy images confirm the suppression of LPS-mediated NETosis by SP-D. **a**–**b** Neutrophils were activated for 2 h by LPS (**a**) or ionomycin (**b**) with or without SP-D at two calcium concentrations and imaged after immunostaining for myeloperoxidase (MPO; green) and staining with DAPI for DNA (blue). MPO co-localized to NET-DNA upon stimulating neutrophils with LPS or ionomycin. **a** Pre-incubation of the indicated concentrations of SP-D with LPS showed the suppression of NETs release. **b** SP-D did not suppress NETosis induced by ionomycin (*n* = 2, with the whole experiment repeated twice, scale bar, 20 μm).

To visualize and confirm the effect of SP-D on LPS-mediated NET formation, we immunostained the neutrophils, that were treated with or without LPS in the absence or presence of SP-D, for the detection of NET markers, myeloperoxidase (MPO) and DNA. Confocal microscopy images of neutrophils stained for DNA (DAPI: 4′,6-diamidino-2-phenylindole; blue) and MPO (green) confirmed that LPS induces NETosis, while SP-D suppresses LPS-mediated NETosis (Fig. 2). Neutrophils stimulated with 5 μg/mL LPS showed decondensed nuclei and DNA strings colocalized with MPO, confirming the formation of NETs. By contrast, the control conditions (unstimulated neutrophils) showed intact nuclei with MPO granules in the cytoplasm. In the presence of LPS and SP-D, NETs were almost absent and neutrophils morphology was similar to that of control cells. No differences were observed in the suppressive effect at the two calcium concentrations tested (Fig. 2a). Furthermore, based on the neutrophil morphology and nuclei decondensation, there is no evidence for neutrophils undergoing apoptosis/necrosis during the suppression of LPS-mediated NETosis by SP-D. Neutrophils treated with ionomycin did not show any response to SP-D, and similar amounts of NETs were observed in the presence or absence of SP-D (Fig. 2b). More cells in low magnification images of the same panel in Fig. 2 are shown in the Supplementary Fig. 5.

Collectively, these data confirmed the induction of NETosis by LPS and the suppressive effect of SP-D only in LPS-mediated NETosis, but not in the NETosis induced by other ligands such as PMA or ionomycin (supplementary Figs. 4–5; Fig. 2).

### SP-D suppresses LPS-mediated citrullination of histone 3

**Fig. 3:**
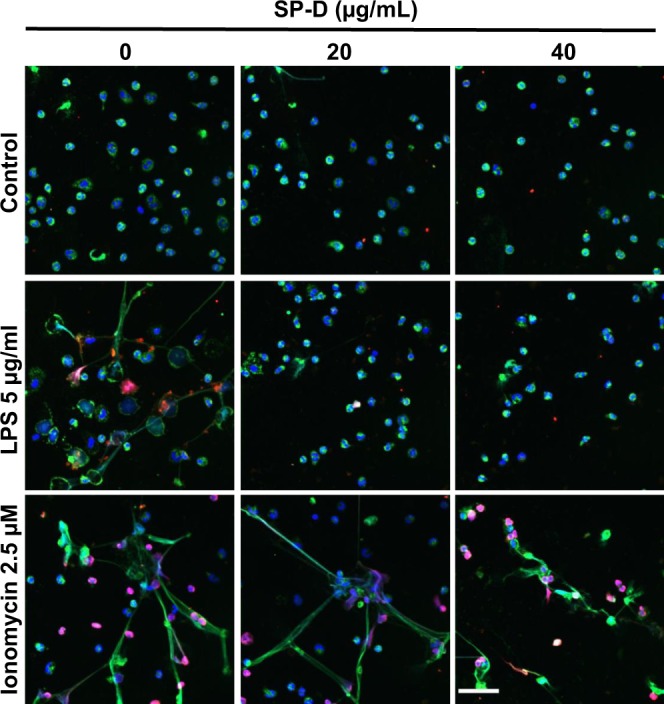
LPS (O128:B12) induces citrullination of Histone 3 (citH3) during NETosis and SP-D suppresses LPS-induced citH3 formation. Neutrophils were activated for 2 h with −ve control (media only), LPS or ionomycin with or without 20 and 40 μg/mL of SP-D. The cells were fixed and immunostained imaged for citH3 (red) and myeloperoxidase (MPO; green) and stained with DAPI for DNA-blue. LPS and ionomycin, but not buffer control, induced citH3 formation that co-localized with DNA and MPO. SP-D suppressed LPS-mediated, but not ionomycin-mediated citH3 formation (*n* = 2, scale bar, 40 μm).

Certain agonists can induce PAD4-mediated citrullination of histone 3 to facilitate NETosis^10,11,14,34^. Citrullination of histone 3 (citH3) is an important regulatory factor in NOX-independent NETosis though some studies also reported the involvement of citH3 in NOX-dependent NETosis. CitH3 being a regulatory factor during NETosis, we were curious to know the potential of LPS O128:B12 to induce citrullination of histone 3 during NETosis in the presence and absence of SP-D. The neutrophils activated with LPS in the presence or absence of SP-D immunostained for citH3 (red) and imaged by confocal microscopy. Ionomycin was used as a positive control (a well-known agonist that induces CitH3). While staining was very low in the untreated control condition, neutrophils treated with LPS exhibited more immunostaining of citH3 (Fig. 3), although the staining level was slightly lower than in the case of ionomycin (positive control). Pre-incubation of LPS with SP-D showed reduced citH3 immunostaining, indicating that the suppressive effect of SP-D occurs during the early stages of NETosis. For single channel and merged images, see the Supplementary Fig. 6. Interestingly, the citH3 suppressive effect of SP-D was not observed towards ionomycin treated cells (Fig. 3). Therefore, both LPS O128:B12 and ionomycin induce citH3 in neutrophils, while SP-D only suppresses citH3 during LPS-, but not the calcium ionophore-induced NETosis.

### LPS instillation induces more NET formation in SP-D^−/−^ mice

**Fig. 4:**
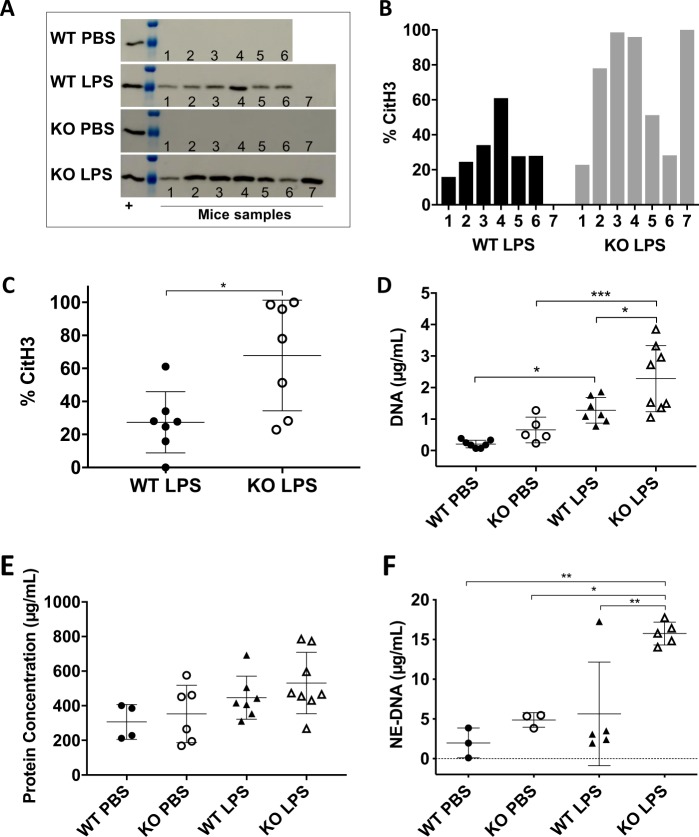
LPS instillation induces exacerbated DNA, CitH3, and NE-DNA in the airways of SP-D^−/−^ KO mice. **a–d**, **f**, Higher amounts of NETs are present in the BAL of KO mice instilled with LPS compared with the WT counterpart, as determined by (**a**) Western blot analyses of citH3 (complete membranes are shown in Supplementary Fig. 7) (**b**) with densitometry of individual samples (**c**) and average data; WT-PBS (*n* = 6), KO-PBS (*n* = 7), WT-LPS (*n* = 7), KO-LPS (*n* = 7); (Two-tailed *t*-test t = 3.06; *p = 0.0222) and (**d**) cell free DNA concentration quantified by PicoGreen assay; WT-PBS (*n* = 7), KO-PBS (*n* = 7), WT-LPS (*n* = 5), KO-LPS (*n* = 8); (One-way ANOVA with Tukey’s multiple comparison post-test, F(3,23) = 14.37, *p* < 0.0001; *p < 0.05; ***p < 0.001). **e** Total protein concentrations in BAL for the different mouse groups; WT-PBS (*n* = 4), KO-PBS (*n* = 6), WT-LPS (*n* = 7), KO-LPS (*n* = 8); (One-way ANOVA with Tukey’s multiple comparison post-test, F(3,21) = 2.62, *p* = 0.0776). **f** Neutrophil Elastase-DNA (NE-DNA) concentration in BAL from the different mouse groups (*n* = 3-5) (One-way ANOVA with Tukey’s multiple comparison post-test, F(3,12) = 9.969, *p* = 0.0014; *p < 0.05, **p < 0.01). Data are represented by individual data points and mean ± SD.

**Fig. 5:**
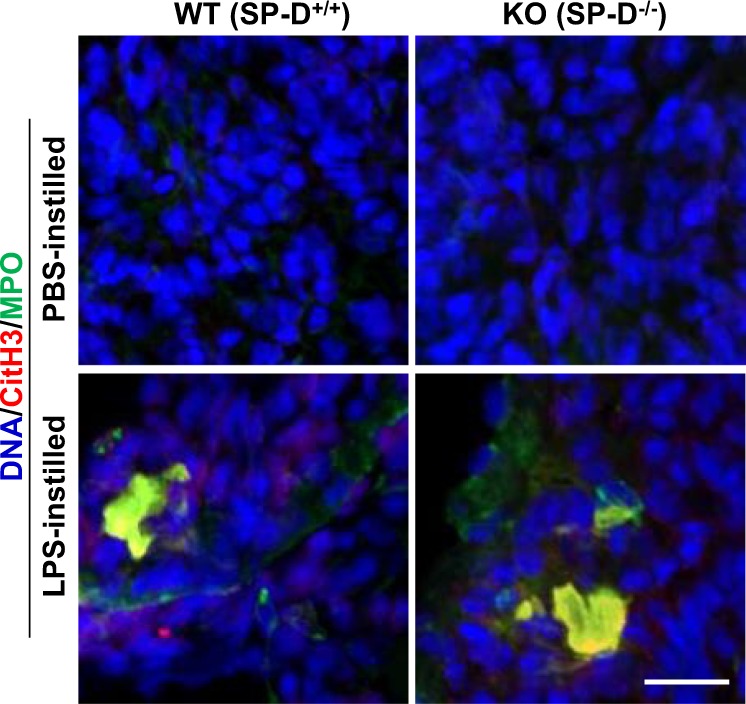
LPS-induced NETosis is high in the lungs of KO mice compared to WT mice. Lung tissue sections were obtained from KO and WT mice that were instilled with either PBS or LPS. Tissue sections were processed, immunostained and imaged for citH3 (red) and myeloperoxidase (MPO; green) and stained with DAPI for DNA (blue). NETosis was absent in mice instilled with PBS. KO mice instilled with LPS exhibited more NETs in lung tissue, compared to the WT mice instilled also with LPS. (*n* = 2; scale bar, 23 μm).

To address whether SP-D could regulate NETosis during LPS-mediated lung inflammation, in vivo, we instilled LPS O128:B12 into the airways of both SP-D^−/−^ KO (knock-out) and SP-D^+/+^ wild type (WT) mice. The BAL samples from these mice were examined for the presence of DNA and citH3 to assess the presence of NETs. Neutrophil elastase-DNA sandwich (NE-DNA) ELISA was also performed to quantify the NETs under these conditions. Furthermore, frozen lung tissue sections were obtained from different groups of mice (KO and WT instilled with LPS or PBS) to perform the immunodetection of NETs in the lung tissue. Significantly higher citH3 was found in samples obtained from LPS-instilled KO mice than in those from the WT counterparts (Fig. 4a–c, Supplementary Fig. 7). PicoGreen DNA-binding fluorescence dye was used for quantifying the DNA present in the BAL supernatant. LPS-instilled mice contained higher amounts of DNA in the BAL supernatant compared to that of the PBS-instilled mice (Fig. 4d). Moreover, the DNA concentrations were higher for LPS-instilled KO mice than for WT counterparts. Total protein concentration determined by BCA assay, showed no substantial differences in total protein concentrations between LPS-instilled KO and WT mice (Fig. 4e; *p* = 0.0776). In addition, quantifying NETs by the NE-DNA based ELISA revealed a significantly higher amount of NETs in LPS-instilled SP-D KO mice BAL samples (Fig. 4f) compared to the other groups. Immunostaining of MPO, CitH3, and DNA in frozen lung tissue sections demonstrated more NETs (colocalization of citH3, MPO and DNA staining), in the lungs of the LPS-instilled KO mice compared to the other groups (Fig. 5). Collectively, these in vivo data (Figs. 4 and 5) suggest that LPS (O128:B12) induces NETosis in mouse airways and show that LPS-induced NETosis is reduced in mouse lungs expressing SP-D.

### Airway inflammation leads to LS dysfunction in SP-D^−/−^ mice

**Fig. 6:**
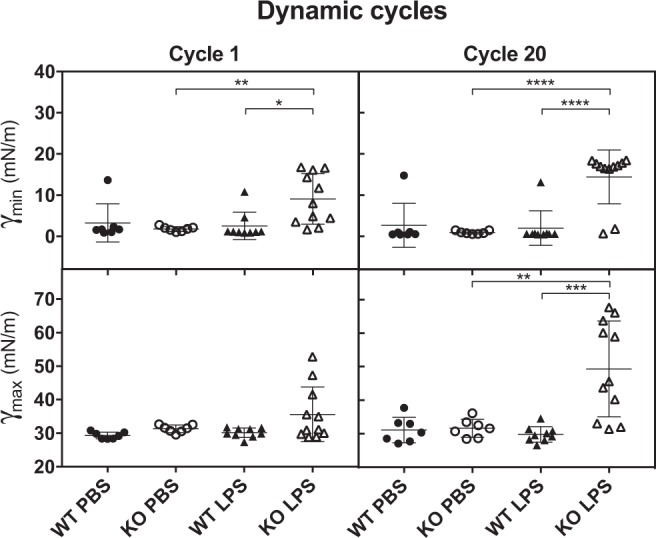
Pulmonary surfactant of LPS-instilled SP-D^−/−^ (KO) mice show surfactant dysfunction. Minimal (γ_min_) and maximum (γ_max_) surface tensions of LS (10 mg/mL) from the different mouse groups during compression-expansion cycling. Symbols represent data obtained from individual mouse (average of three replicates). WT-PBS (*n* = 7), KO-PBS (*n* = 7), WT-LPS (*n* = 9), KO-LPS (*n* = 11); (One-way ANOVA with Tukey’s multiple comparison post-test, Cycle 1 (γ_min_) F(3,30) = 5.547, *p* = 0.0038; Cycle 20 (γ_min_) F(3,30) = 15.95, *P* < 0.0001; Cycle 1 (γ_max_) F(3,30) = 3.275, *p* = 0.0346; Cycle 20 (γ_max_) F(3,30) = 11.62, *p* < 0.0001; post-test **p < 0.01; ***p < 0.001; ****p < 0.0001). Supplementary Fig. 8: initial adsorption of LS of all the experimental mouse groups. Supplementary Fig. 9: individual examples of the CBS isotherms for individual mouse of all experimental groups.

At baseline condition, SP-D deficiency in mice does not affect the surface-active properties of lung surfactant^35,36^. However, the importance of SP-D in maintaining the biophysical properties of lung surfactant during inflammation has not been established. Therefore, the biophysical activity of lung surfactant (LS) obtained from BAL was evaluated in the captive bubble surfactometer (CBS). The CBS setup mimics the in vivo situation of a single alveolus during breathing. LS is applied onto the air-liquid interface of an air-bubble inside a chamber filled with buffer, which upon adsorption of the surfactant, can be compressed and expanded like the alveolus is in the lung. Adsorption of the surfactant of all the experimental groups was excellent; no differences were observed among the different mouse groups (Supplementary Fig. 8). When the surfactant interfacial layer underwent repetitive compressions-expansions (dynamic cycles, 30 cycles/min), all the surfactants, except the one from LPS-instilled SP-D KO mice, reached very low minimum surface tensions <2 mN/m while maintaining maximum tensions around 30 mN/m (Fig. 6), as one would expect for a well-functioning lung surfactant^37^. Interestingly, LS function was seriously compromised in KO mice instilled with LPS. LS of KO mice with LPS-mediated lung inflammation was unable to reach the low surface tensions during compression that are required for efficient breathing. Also, the maximum surface tensions of these surfactants were high in comparison to other mouse groups (see examples of the CBS isotherms for individual mouse samples in Supplementary Fig. 9). Considering these data, we conclude that compared to the WT mice, biophysical activity of surfactant of SP-D-deficient mice is compromised during lung inflammation.

### SP-A, SP-C and cholesterol do not affect LS dysfunctions

**Fig. 7:**
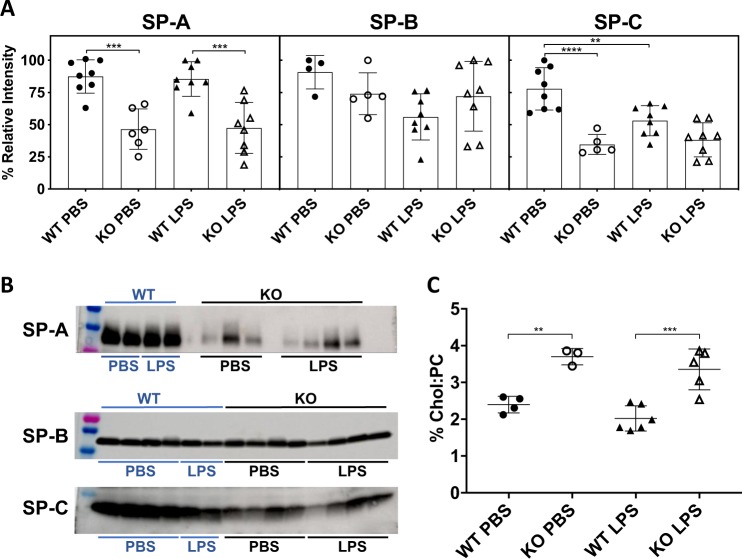
SP-A, SP-B and SP-C concentrations, and cholesterol content in the surfactant isolated from SP-D^+/+^ (WT) and SP-D^−/−^ (KO) mice. **a** Densitometry of the bands of surfactant proteins revealed in Western blots for LS samples from all mice groups; SP-A: WT-PBS (*n* = 8), KO-PBS (*n* = 8), WT-LPS (*n* = 6), KO-LPS (*n* = 8), One-way ANOVA with Tukey’s multiple comparison post-test F(3,26) = 15.78, *p* < 0.0001; SP-B: WT-PBS (*n* = 4), KO-PBS (*n* = 8), WT-LPS (*n* = 5), KO-LPS (*n* = 8), One-way ANOVA with Tukey’s multiple comparison post-test F(3,21) = 2.659, *p* = 0.0746; SP-C: WT-PBS (*n* = 8), KO-PBS (*n* = 8), WT-LPS (*n* = 5), KO-LPS (*n* = 8), One-way ANOVA with Tukey’s multiple comparison post-test F(3,25) = 16.17, *p* < 0.0001. Graph **p ≤ 0.01; ***p ≤ 0.001; ****p < 0.0001. **b** Representative Western blots for SP-A, SP-B, and SP-C from the different mouse groups, complete membranes are shown in Supplementary Fig. 10. **c** [cholesterol]/[phosphatidylcholine] ratio, WT-PBS (*n* = 4), KO-PBS (*n* = 3), WT-LPS (*n* = 6), KO-LPS (*n* = 5), One-way ANOVA with Tukey’s multiple comparison post-test F(3,25) = 16.17, *p* < 0.0001; **p ≤ 0.01; ***p ≤ 0.001.

Differences in the levels of major surfactant proteins SP-A, SP-B, and SP-C may affect the surface activity of surfactant^37^. Therefore, we analyzed the levels of these proteins present in the LS of the four different mouse groups by Western blotting (Fig. 7a, b, Supplementary Fig. 10). Results showed that KO mice had lower levels of proteins SP-A and SP-C than WT mice while SP-B levels were comparable in both SP-D-sufficient and SP-D-deficient mouse surfactant. Only SP-C levels were significantly lower when the WT mice were instilled with LPS instead of PBS. However, no significant changes were observed for any of the proteins as a consequence of LPS instillation in KO mice.

Previous studies showed that phospholipid composition of lung surfactant from SP-D-deficient and WT mice do not differ significantly^26,36^. However, cholesterol content could affect the surface properties of surfactant^22,38^. Therefore, we determined the cholesterol concentration of LS. Slightly higher proportions of cholesterol compared to PC were found in KO mice (3.3–3.7%) than in WT mice (2-2.4%), but LPS-mediated inflammation did not change the ratio (Fig. 7c). Therefore, the changes in other surfactant proteins and in the cholesterol/PL ratio would not explain the differences seen in surface activity that was compromised only in the SP-D KO mice during LPS-mediated inflammation.

### SP-D protects surfactant from NET-mediated inhibition

**Fig. 8:**
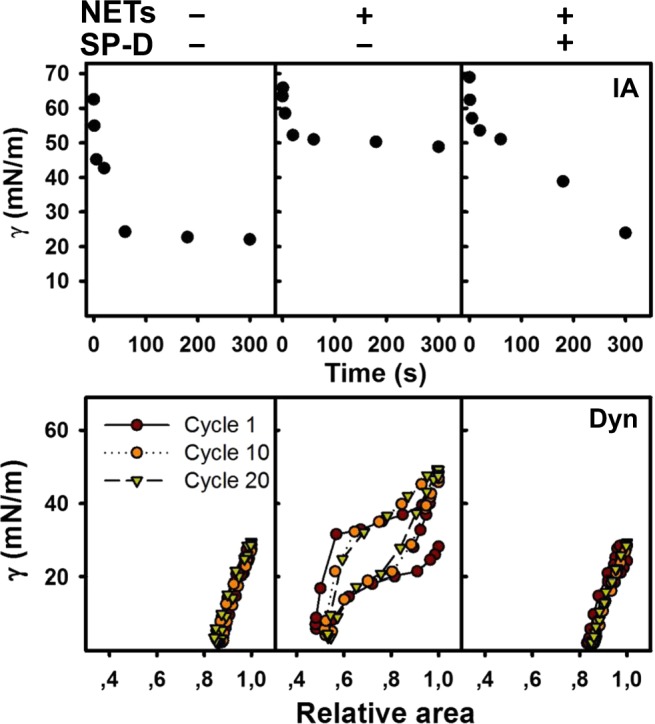
NETs inhibits surface-active properties of pulmonary surfactant, and SP-D mitigates the inhibitory effect of NETs on surfactant. Surface activity is assessed by the isotherms obtained from the CBS, thermostated at 37 °C. Representative isotherms from each group of samples are shown. Surfactant films were formed upon injection of a suspension of surfactant (20 mg/mL) with or without NETs, in the absence or presence of SP-D (0.67% w/w with respect to phospholipid). The upper set of isotherms show IA to the air-water interface of a bubble over a 5-min period. The lower set of isotherms illustrates dynamic compression-expansion cycling (at 20 cycles/min) for cycles 1, 10 and 20. The detail parameters are enlisted in Supplementary Table 1.

Whether NETs could directly inhibit the surface-active properties of surfactant is unknown. Therefore, as the final step, we conducted experiments to determine whether NETs could be directly responsible for the inactivation of surfactant function, and whether SP-D could prevent such an inhibition. Organic extract (OE) of lung surfactant purified from porcine lungs, which is depleted of SP-A and SP-D, is used as the basic component of clinical surfactants^22^. We used this surfactant as a model to study the direct effect of NETs on the biophysical function of LS. Reconstituted OE material was combined with purified NETs obtained from neutrophils and its biophysical activity was evaluated in the CBS. NETs completely inhibited the surfactant function, even though the concentration of NETs applied was lower than the ones found in vivo in the BAL of KO LPS-instilled mice. As expected^37^, surface tension of the control surfactant reached ~20 mN/m after initial interfacial adsorption (IA). By contrast, in the presence of NETs, surface tension after adsorption was still high, in the order of ~50 mN/m (Fig. 8 and Supplementary Table 1). The addition of SP-D fully overcame the inhibitory effect of the NETs as observed in our experiments and brought the surface tension to values comparable to those produced by surfactant without the NETs.

NETs also affected the dynamic behavior of surfactant, and minimum and maximum surface tensions reached during dynamic cycles were high, far from the minimal values of ≤2 mN/m and ~25 mN/m, respectively, typically produced by a good surfactant film. SP-D was also found to counteract the inhibitory effect of NETs on the dynamic behavior of surfactant (Supplementary Table 1). These experiments collectively showed that NETs directly inhibit the biophysical function of lung surfactant, and that SP-D can protect LS against the surface tension inhibitory effects of the NETs.

### NETs impair lateral packing of lung surfactant membranes

**Fig. 9:**
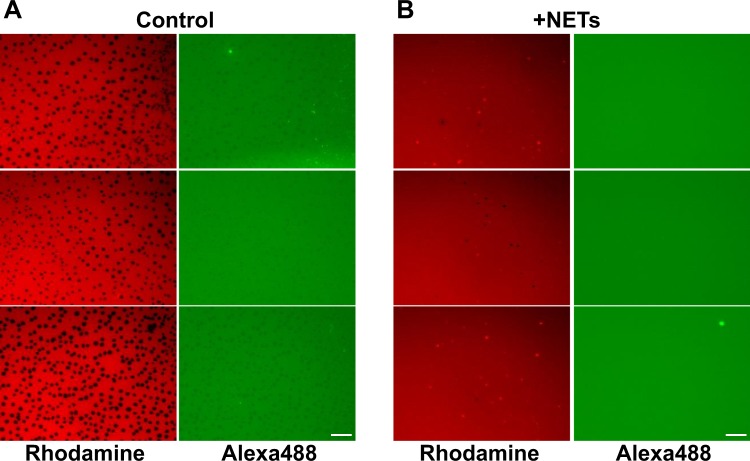
NETs impair the structure and lateral packing of phospholipids in interfacial lung surfactant films. Lung surfactant was labeled with a trace of fluorescent Alexa488-labeled surfactant protein SP-B (F-SP-B, 1% (w/w) with respect to lipids) and with rhodamine-DOPE (0.1% mol/mol probe/phospholipid) in the presence or absence of purified NETs. The surfactant was then applied onto the air-water interface of a Langmuir surface balance and the films formed were compressed to a surface pressure of 30 mN/m. The compressed films were finally transferred to glass cover slips and analyzed under the epifluorescence microscope. **a** In the absence of NETs (control), compressed lung surfactant films segregate probe-excluding highly-packed domains enriched in saturated phospholipids, which are necessary to reach the lowest surface tensions. **b** In the presence of NETs, condensed phospholipid domains are not observed, being the structure and lateral packing of the lung surfactant film severely impaired. Scale bar, 25 μm.

To determine how NETs affect lung surfactant biophysical function, the structure of the LS film subjected to compression at the air-water interface was observed in the presence and absence of NETs. Experiments were conducted in a Langmuir surface balance, doping surfactant OE with a trace of unsaturated phospholipids and SP-B that were fluorescently labeled with Rhodamine-DOPE and Alexa488, respectively. Figure 9 shows how NETs induce a profound alteration of the structure of the LS film at the air-water interface. Under control conditions (absence of NETs, Fig. 9a), surfactant films exhibited good compressibility, with compression inducing the segregation of probe-excluding highly packed domains (black regions) likely enriched in saturated phospholipids. Condensed domains are required to achieve low surface tensions during surfactant film compression^23^. However, in the presence of NETs (Fig. 9b), these domains were not observed in surfactant films subjected to compression, indicating that the LS films were not able to properly sort saturated from unsaturated phospholipid species at the air-water interface. The absence of tightly packed phospholipid domains competent to provide maximal stability at very highly compressed states correlates with the inability of these films to reach low enough surface tensions. No differences were observed in the apparent distribution of SP-B between the two conditions. Fluorescently-labeled SP-B appeared homogenously distributed within the expanded regions of the films. Thus, exposure to NETs affects the re-organization of phospholipids that occurs during the compression of surfactant films, and that is required for the films to reach the maximal packing associated with the minimal surface tensions.

## Discussion

SP-D plays an important role in the immune defense of the lungs and in LS homeostasis^25^. Studies have shown the involvement of NETs during lung inflammation^30,39,40^. Douda et al. have shown that SP-D directly interacts with NETs and bacterial surfaces, simultaneously^30^. However, whether binding of SP-D to LPS would suppress LPS-mediated NETosis was unknown. Also, whether NETs could inhibit surface tension-lowering properties of surfactant and the ability of SP-D to protect NET-mediated surfactant inhibition was also unknown. In this study, we have demonstrated that (i) SP-D dose-dependently attenuates LPS (O128:B12)-induced NETosis; (ii) NETs inactivate lung surfactant, and (iii) SP-D can protect surfactant from NET-mediated inactivation during breathing-like interfacial mechanics. Therefore, we have identified novel functions for SP-D:LPS, SP-D:NET, and SP-D:lung surfactant interactions and revealed the mechanistic relevance of these interactions in regulating airway-related pathobiological functions.

Palaniyar et al. identified the interaction between several collectins, including SP-D, and DNA^29^. Douda et al. further showed that SP-D avidly binds to NETs, and interconnects bacteria with NETs^30^. These studies suggest that the collagenous domains of SP-D interact with DNA and NETs whereas globular lectin domains bind bacteria. It has been well established that SP-D binds to several carbohydrate ligands, including LPS from different strains of bacteria via its lectin domains^28,33^. Previous studies have shown that LPS from different bacterial strains induce NETosis to different degrees^6,41,42^. Our data show that SP-D recognizes LPS (O128:B12) in a calcium-dependent manner (Fig. 1a), which is a hallmark of SP-D recognizing carbohydrate ligands via its lectin domain^31,43,44^. LPS (O128:B12) induces NETosis in a dose-dependent manner, and when this LPS was pre-incubated with SP-D, SP-D dose-dependently inhibit the LPS-mediated NETosis (Fig. 1b–d). This effect is specific for LPS, and SP-D does not inhibit the NETosis induced by other agonists (e.g., PMA, ionomycin). This inhibitory effect is confirmed by immunofluorescence microscopy (Fig. 2). In addition, both the doses and strains of LPS can affect the level of NET formation^6,45,46^. This LPS (O128:B12) also induces citH3 formation that is useful for some forms of NETosis, and this effect is also prevented upon binding of SP-D to LPS (Fig. 3). Therefore, SP-D binds to LPS and inhibits LPS-mediated NETosis.

Previously, a mouse model has been established to study the NETosis in vivo^30,47^. Saffarzadeh and colleagues reported the presence of NETs in lung sections from mice in a model of lung injury induced by LPS. Co-localization of NE, histones and DNA was shown by immunostaining of the lung sections in WT mice^47^. Another study reported the increased level of SP-D in the BAL of WT mouse (instilled with LPS), compared to WT mouse instilled with PBS. This increase in SP-D concentration was associated to the increase in neutrophils infiltration into the airways^30^, indicating a probable direct relationship between SP-D, neutrophils and NETosis. When LPS (O111:B4) was instilled into the airways of mice, neutrophil count peaked at 1–2 days post instillation. When LPS (O128:B12) was instilled, we could see a clear difference in the NETosis levels on day 1-post LPS instillation between WT and SP-D KO mice (Figs. 4 and 5). SP-D KO mice generated more NETs, as determined by NE-DNA, immunostaining of frozen lung sections, citH3 and DNA levels. Therefore, SP-D deficiency is associated with increased NETosis in vivo. This finding is consistent with the ability of SP-D to bind LPS and suppress NETosis, ex vivo (Figs. 1–3). It is important to note that the lack of SP-D in the KO animals is associated with a somehow higher intrinsic pro-inflammatory status. Therefore, the higher level of neutrophil infiltration and NETosis could be a consequence of many factors beyond SP-D deficiency. However, previous studies showed that SP-D levels increase after LPS instillation^30^; hence, the contribution of SP-D-mediated suppression of LPS-induced NETosis may even be enhanced in vivo. Notably, inflammatory lung diseases such as acute respiratory distress syndrome or, cystic fibrosis are associated with exacerbated NETosis, and have low levels of SP-D in the airways^1,25,47–49^. Therefore, lack of enough SP-D might be an additional factor to increase the deleterious effect of inflammation in some respiratory pathology, as a consequence of possibly exacerbated NETosis.

The negative effects of excess NET formation upon lung inflammation and injury have been highlighted by us and others^1,16,47,50,51^. One of the previous studies showed that NETs may be a contributor to the reduction in lung function and compliance in a double hit model (mechanical ventilation + O111:B4 LPS)^52^. However, whether NETs could impair lung surfactant biophysical function was unknown. The present data show that SP-D deficiency in mice results in a severe surfactant inactivation during LPS-mediated inflammation (Fig. 6). In the presence of SP-D (WT mice) instillation of LPS did not show apparent effects on the biophysical behavior of LS isolated from these mice. Probably, higher doses of LPS to induce greater NETosis and acute lung injury, or longer times than 24 h, are needed to see a perturbation in the LS biophysical behavior in WT mice, compared with the conditions selected in this study, which are the described in a previous in vivo model of NETosis^30^. In vitro experiments conducted with surfactant lipids and different concentrations of SP-A suggested a potential role for SP-A in regulating surface active properties of surfactant^53^. However, studies conducted with SP-A-deficient mice and using higher concentrations of surfactant lipids relevant to the context of airways established that SP-A does not have a significant role in regulating surface tension or protecting surfactant from inactivation by various inhibitors^54–56^. Nevertheless, we have analyzed the levels of SP-A in the surfactant from the WT and SP-D deficient mice (Fig. 7). SP-A levels were lower in the SP-D deficient mice, as previously reported^35^, but SP-A levels did not change during LPS-mediated inflammation (Fig. 7). Therefore, SP-A is less likely to contribute directly to the effects seen in our in vivo and in vitro studies. SP-B and SP-C are the surfactant proteins with more direct participation in surface tension reducing properties of surfactant^23,37^. Their levels also would not explain the differences in the surface activities seen in our studies, because the levels of these proteins were comparable in the KO mice exposed to PBS and LPS conditions.

Cholesterol/PL ratio is another factor that could affect surface tension and that has been identified as a pathogenic factor in lung diseases^22,38^. We found that the ratio is higher in the surfactant isolated from SP-D-deficient mice (Fig. 7c). However, the ratio is similar in the LS with or without LPS-mediated inflammation. Moreover, cholesterol concentrations in KO mice were lower than the concentrations that have been reported to cause surfactant inhibition^38^. Therefore, cholesterol content does not seem to be a contributory factor to LPS-induced surfactant dysfunction. Serum proteins have been reported to inhibit LS^57^, but total protein concentrations in BAL from our LPS-instilled mice were low compared to the concentrations that were reported to cause inhibition^58^. Therefore, although other factors (e.g., ROS, pro-inflammatory mediators) may contribute to LS dysfunction, based on our results we propose that NETs directly inhibit LS biophysical function, at least in this in vivo model. Alterations in surfactant composition as a consequence of the lack of SP-D (and the concomitant lack of an appropriate recycling and renewal of spent surfactant^35,59^), could also lead to a complex that could be more susceptible to inactivation, by NETs and/or by other inhibitory substances. Lack of SP-D in vivo could then simultaneously contribute to several surfactant-related pathogenic factors, either directly—as a consequence of the lack of protection imparted by SP-D binding- or indirectly—via the alteration of surfactant composition and structure resulting from impaired recycling.

Nevertheless, our complementary in vitro studies identified a direct inhibitory effect of NETs in LS performance, which can be prevented by the mere presence of SP-D (Fig. 8). This inactivation is associated with a profound alteration of the lateral structure of interfacial surfactant films that is competent to produce the lowest surface tensions at the end of compression. Exposure to NETs prevents the compression-driven segregation of condensed domains, presumably enriched in saturated phospholipid species, which are the hallmark of functional lung surfactant films^60–62^ (Fig. 9). It is plausible that the inhibition of LS biophysical behavior could be mediated by the DNA component of NETs, which might disrupt the lateral packing of surfactant membranes and the organization of the different protein and lipid species at the interface, particularly those charged at physiological pH, as a consequence of its polyanionic character. Therefore, the ability of SP-D to interact with DNA^29^ could be important for its direct protective effect, besides its anti-inflammatory actions mediated via LPS scavenging. Interestingly, we found comparable levels of surfactant inactivation by different preparations of NETs that were always dosed considering the DNA content, supporting that DNA is the most active component with regard to surfactant inhibition.

**Fig. 10:**
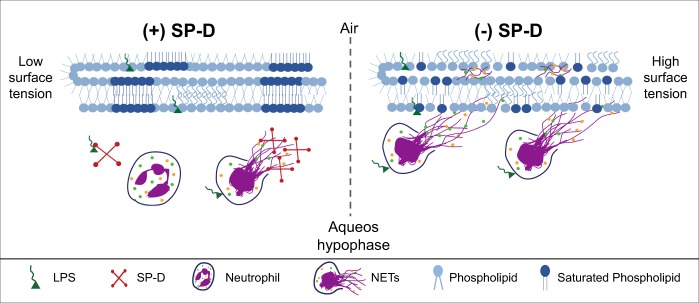
A model summarizing the effect of SP-D on LPS-mediated NETosis and NET-mediated inhibition of surfactant. In the presence of SP-D (+SP-D), SP-D sequesters LPS and suppresses LPS-mediated induction of NETosis; any NETs formed by the neutrophils will be coated with SP-D. These two effects of SP-D would suppress NET formation and NET-mediated inactivation of lung surfactant. In this case, lung surfactant would present a functional structure, exhibiting saturated phospholipid domains (dark blue), which are necessary to reach very low surface tensions when the monolayer is compressed. In the absence of SP-D (−SP-D), LPS would directly interact with neutrophils and induce NETosis. NETs released from these neutrophils will inhibit the surface-active properties of the surfactant, disrupting the phase separation and organization of phospholipids at the interface, being unable to form highly packed domains. Our data sets indicate that SP-D significantly contributes to the protective effects seen in our in vitro, ex vivo, and in vivo experiments.

Considering all the data, we propose a key role for SP-D in modulating NETosis and contributing to protect the lung and the pulmonary surfactant system. SP-D binds to LPS and reduce LPS-mediated NETosis. In the absence of SP-D, neutrophils might undergo NETosis to a greater extent that results in higher amounts of NET accumulation at the alveolar spaces and its interaction with and perturbation of the surfactant layer. NET-surfactant interactions could lead to a profound disruption of the multilayered surfactant structure and a subsequent impairment of the surface active functions of the surfactant. When SP-D is present, interactions between the NET and SP-D could reduce NET-mediated inactivation of surfactant (Fig. 10). Therefore, SP-D could play a dual protective role, as an important inhibitor of LPS-mediated NET formation and as an inhibitor of NET-mediated surfactant inactivation during lung inflammation. It is important to consider though that the actual scenario in vivo has additional complexities. Differences in the level of SP-D could not only have consequences on the level of NETosis in the presence of LPS, but as a consequence of different processes related with the higher pro-inflammatory contexts associated to low levels of the collectin. Low SP-D level scenarios also imply defective surfactant recycling and potentially accelerated compositional and functional impairing, which may results in surfactant complexes that are intrinsically more susceptible to inactivation by many agents including NETs. Total or partial inactivation of surfactant, and the subsequent defective breathing mechanics, is a recognized additional lung pro-inflammatory contribution, which may induce further compromise of alveolar homeostasis. Hence, SP-D may be a potential therapeutic candidate for minimizing surfactant inhibition in inflammation-related multi-factorial respiratory pathologies.

## Methods

### Ethics statement

Human and animal study protocols were approved by the Research Ethics Board (REB) of the Hospital for Sick Children, Toronto, Canada. Donors signed the consent form prior to the participation. In addition, all animal protocols were approved by the Toronto Centre for Phenogenomics (TCP) (Toronto, Canada), and all the procedures were performed according to the protocols and ethical guidelines.

### Human peripheral neutrophil isolation

Peripheral blood from healthy male donors was collected in K2 EDTA blood tubes. We exclude blood collection from any healthy donor having flu or any known inflammatory symptoms. Isolation of neutrophils was carried out as described previously^6,46,63^. Briefly, PolymorphPrep (Axis-Shield) was used for isolating polymorphonuclear neutrophils from blood. Red blood cells were then lysed and cellular debris were eliminated using consecutive washes as stated previously (see references for details). Purified neutrophils were resuspended in RPMI medium (Invitrogen) containing 10 mM HEPES (pH 7.2). Cells were counted and viability was checked by Trypan blue using a hemocytometer. Purity of neutrophils was determined by Cytospin preparation and imaging. Neutrophil preparations with >95–98% pure viable cells were used in this study.

### NETs preparation and purification

Human neutrophils (100,000) in 1 mL of RPMI media were stimulated with 5 μg/mL LPS (O182:B12) from *Escherichia coli* (Sigma) to induce NETosis. Samples were incubated for 4 h at 37 °C in 5% CO_2_ with intermittent gentle mixing by inverting the tubes (once each 30 min), in order to avoid cell sedimentation. After incubation, samples were centrifuged at 400×*g* for 10 min. Supernatants were collected and snap-frozen in dry ice. DNA concentration in the purified NETs was determined with Quant-iTPicoGreen dsDNA Kit (Invitrogen) following manufacturer´s instructions.

### SP-D purification

Human SP-D was obtained from therapeutic bronchoalveolar lavage (BAL) of individuals with pulmonary alveolar proteinosis. The purified hSP-D from these BALs has been used in many functional studies as reported earlier^64,65^. Briefly, BAL was incubated with maltose-agarose beads in the presence of 10 mM CaCl_2_. The beads were poured into an empty column and washed with 1 M NaCl to remove non-specifically bound components on an AKTA FPLC system (G.E. Healthcare). SP-D was eluted with Tris-MnCl_2_ buffer (20 mM Tris (pH 7.4), 100 mM MnCl_2_). Then, fractions containing SP-D were pooled and concentrated, and further purified with a Superose 6 (10 × 300 mm) gel filtration column in 20 mM Tris (pH 7.4), 150 mM NaCl, 5 mM EDTA buffer, as previously described^64,65^. The oligomeric structural intactness of the purified SP-D from the BAL of proteinosis patients has been assessed by electrophoresis and atomic force microscopy (AFM)^27^. The AFM images show that hSP-D purified from proteinosis preserves its typical oligomeric structure, assembled as large oligomers (Supplementary Fig. 1). Routine functional experiments confirm that this protein preparation retains the ability to bind and agglutinate bacteria (i.e., *E. coli*).

### SytoxGreen NETosis assay

SytoxGreen (Life Technologies), a cell-impermeable nucleic acid binding dye, was used for monitoring NETosis over time. Microplates of 96-wells were seeded with 100 μL of a suspension containing 50,000 neutrophils and 5 μM SytoxGreen dye in each well. Agonists (known NETs inducers; positive control), or agonists with SP-D (at 10, 20, 40 μg/mL), or RPMI with SP-D buffer (negative control) were prepared in 10 μL of media and incubated for 30 min at 37 °C in 5% (v/v) CO_2_ incubator. Calcium concentration of SP-D buffer was adjusted prior to use at 5 mM CaCl_2_. After incubation time, a volume of 10 μL, suspension containing buffer alone (negative control), or agonist (with and without SP-D) was added to the cells and the fluorescence intensity of the dye was tracked at 30-min time intervals up to 240 min after neutrophil activation, using a POLARstar OMEGA fluorescence plate reader. Between readings, the plates were kept at 37 °C in 5% (v/v) CO_2_ incubator. Final calcium concentration in the experiment was 0.5 mM. The final concentrations of the different agonists in these experiments were 25 nM PMA, 2.5 μM ionomycin (calcium ionophore produced by the bacterium *Streptomyces conglobatus*) and 5 μg/mL LPS. For LPS dose-response, different LPS concentrations were titrated. The background fluorescence at 0-min time point was subtracted from the fluorescence at each time point and was then divided by the fluorescence values of the neutrophils lysed with 0.5% (v/v) Triton X-100 (the maximum fluorescence in the neutrophils treated with Triton X-100, considered as 100% DNA release).

### Immunostaining and confocal imaging

Neutrophils (50,000 in 100 μL of RPMI media/well) were seeded in 12-well chamber slides (Ibidi). The agonists and controls with or without SP-D prior adding to neutrophils were prepared as described above. In this case, two final calcium concentrations (0.5 mM and 2 mM) were tested. After stimulation, neutrophils were placed at 37 °C, 5% CO_2_ incubator for 2 h. Cells were then fixed with 4% (w/v) PFA for overnight at 4 °C. After fixation, samples were washed three times with PBS and permeabilized with 0.05% (w/v) Triton X-100 for 10 min. Cells were washed three times with PBS again. For non-specific antigen blocking, 4% (w/v) BSA were used for 1 h. After blocking, primary antibodies were added to the samples and incubated overnight (ON) at 4 °C. The antibodies used were anti-myeloperoxidase mouse antibody (ab25989, Abcam) at 1:500 for staining myeloperoxidase (MPO) (with anti-mouse secondary antibody conjugated with a green fluorescence Alexa fluor 488 dye; 1:5000 dilution; ThermoFisher), while rabbit anti-citH3 antibody (ab5103; Abcam) at 1:500 dilution was used for detecting the presence of citrullinated histone H3 (CitH3, with anti-rabbit secondary antibody conjugated with a far-red fluorescence dye Alexa fluor 647; 1:5000 dilution; ThermoFisher). DNA was stained with DAPI (4’,6-diamidino-2-phenylindole) (1:100 dilution of the stock 1 mM). After incubation with the secondary antibody, slides were washed and mounted in glass cover slips (ThermoFisher) with anti-fade fluorescent mounting medium (Dako). The images were taken using an Olympus IX81 inverted fluorescence microscope with a Hamamatsu C9100-13 back-thinned EM-CCD camera and Yokogawa CSU × 1 spinning disk confocal scan head with Spectral Aurora Borealis upgrade, four separate diode-pumped solid-state laser lines (Spectral Applied Research, 405, 491, 561, and 642 nm). Samples were imaged at ×40/0.95 magnification and images were processed by Volocity software (version 6.3, Cell Imaging Perkin-Elmer).

### Dot Blot

LPS (O182:B12) dilutions at 2.5, 5, 10, 20, 60, and 80 μg/mL were dotted (2 μL) onto a nitrocellulose membrane (Amersham Biosciences). SP-D (370 ng) and SP-D buffer were also applied as positive and negative controls, respectively. After drying at room temperature (RT), membranes were blocked with 5% (w/v) BSA in TBST-Ca buffer (50 mM Tris (pH 7.4), 150 mM NaCl, 0.02% (v/v) Tween and 5 mM CaCl_2_) overnight at 4 °C. Three washes with TBST-Ca buffer were done and the different membranes were incubated with 5 μg/mL or 15 μg/mL of SP-D in TBST-Ca or 5 μg/mL of SP-D in TBST-EDTA (20 mM) for 5 h at RT. A control set with TBST-Ca without SP-D was also performed. After incubation, membranes were washed four times with TBST-Ca and incubated with SP-D antibody against human recombinant SP-D (custom polyclonal anti-SP-D generated in rabbits by Cocalico Biologicals, PA, USA)^66^ at 1 μg/mL ON at 4 °C, followed by four washes with TBST-Ca. Membranes were then incubated for 1 h with the secondary antibody, donkey anti-rabbit IgG-HRP (ThermoFisher; 1:10000) and washed as described above. SP-D complexes were detected by ECL (Millipore).

### Mice and BAL sample processing

Balb/c wild type (WT) SP-D^+/+^ and Knock out (KO) SP-D^−/−^ mice (generated by Dr. S. Hawgood, UCSF) were bred in the Toronto Centre for Phenogenomics (TCP), Canada. For airway instillation, mice were anesthetized with 2–4% (v/v) isofluorane with O_2_ at 1 L/h. Then, 5 μg of LPS (O182:B12) in 25 μL of sterile PBS or 25 μL of sterile PBS (negative control) were instilled intranasally. Mice were sacrificed with an intraperitoneal injection of 0.1 mL of Euthanyl (Bimeda-MTC) 24 h after instillation, and three times with 1 mL of buffer (Tris 5 mM (pH 7.4), NaCl 150 mM) were used to flush the lungs. All procedures were performed in accordance with TCP approved protocols and ethical guidelines.

BAL samples were frozen with dry ice and stored until use at −80 °C. Prior to use each sample was thawed at 37 °C and centrifuged for 10 min at 400×*g*, discarding the pellet. An aliquot of 400 μL of BAL supernatant was saved and frozen for further experiments (determination of CitH3 and total protein, DNA and NE-DNA ELISA in BAL). Two mililiter of BAL supernatant aliquots were ultra-centrifuged at 100,000×*g* (4 °C, 1 h) to pellet lung surfactant components. Surfactant pellets were resuspended in 10–15 μL of buffer (Tris 5 mM (pH 7.4), NaCl 150 mM). Phosphatidylcholine (PC), as a reference for phospholipids (PL) concentration, and cholesterol in lung surfactant samples were determined using kits (Spinreact) based on enzymatic methods^67,68^. Samples were tested in the captive bubble surfactometer (CBS) at a concentration of 10 mg/mL of PC.

Total protein and DNA concentrations were determined in BAL supernatants. Pierce BCA Protein Assay Kit (ThermoFisher) was employed to determine total protein concentration in the samples. Quant-iTPicoGreen dsDNA Kit (Invitrogen) was used for obtaining DNA concentration in BAL supernatants, and in this case, samples were diluted 1:3, and 50 μL of the diluted samples were assayed. Manufacturer’s instructions for 96-wells plate protocols were followed for both commercial kits.

### Neutrophil elastase (NE)-DNA ELISA

All the BAL samples, from WT and SP-D KO mice instilled with LPS or vehicle control (PBS), were analyzed for neutrophil elastase-DNA (NE-DNA) complex using a sandwich ELISA protocol. A volume of 100 μL of undiluted samples were added in duplicate to a 96-well plates, which were pre-coated with anti-neutrophil elastase antibodies (MyBioSource). The plates were sealed and incubated for 90 min at 37 °C. After the incubation, wells were washed three times by using 350 μL 1× washing buffer. After washing without dehydrating the wells, a volume of 100 μL of anti-DNA antibody was added to each well (diluted 1:100 in incubation buffer; Roche, catalog #11774425001). The plates were again sealed and incubated for 2 h on a rocker at room temperature. After the incubation wells were again washed, and 100 μL of ABTS substrate (MyBioSource, catalog #MBS269576) was added to each well. After substrate incubation for 30 min at room temperature in the dark, the reactions were stopped by adding the termination solution and the absorbance values were read at 405 nm using a plate reader. NETs prepared from human neutrophils with PMA activation were quantified (DNA content) and ran as a control to build a standard-curve.

### Immunolocalization of CitH3 and MPO in frozen lung section

SP-D KO and WT mice were instilled with either PBS control or LPS. After 24 h, lungs were perfused with 1 mL of optimal cutting temperature (OCT) compound (Tissue-Tek; Sakura Finetechnical Co., Ltd., Tokyo, Japan) through trachea. After tying off the trachea to maintain the fluid in the lung, the whole lung was further emerged into the OCT and preserved at −80 °C. Frozen lungs were cut as 10 μm sections in a cryostat microtome and mounted on glass slides. Lung tissue sections from different groups (WT and KO mice, instilled with either PBS or LPS) were thawed at room temperature and fixed in 4% (v/v) paraformaldehyde for 15 min. After washing, sections were treated with 5% (w/v) BSA for 1 h at room temperature to block the nonspecific binding. Rest of the immunostaining steps and confocal imaging were followed as stated above (see section “Immunostaining and confocal imaging”).

### Reconstituted porcine surfactant extract

Organic extract (OE) from native surfactant purified from porcine lungs was obtained as previously described by Schürch and colleagues^37^. A volume of 335 μL of OE at 8.97 μg/μL were evaporated under nitrogen flow and then under vacuum in an UNIVAP at 37 °C, for 2 h to remove traces of organic solvents. Samples were reconstituted to 60 mg/mL final PL concentration in a two-step process: (i) first, 25 μL of buffer 5 mM Tris (pH 7.4), 150 mM NaCl were added and incubated at 45 °C for 30 min; (ii) then, 25 μL of purified NETs in RPMI media with a DNA concentration of 0.65 μg/mL were also added and incubated again at 37 °C for another 30 min. All incubations in the reconstitution process were performed with intermittent shaking (10 min shaking/10 min repose; 1400 rpm in a Thermomixer). For recovering experiments, the 25 μL of buffer in reconstitution step 1 were substituted by 25 μL of SP-D at 808 μg/mL (final SP-D concentration in the sample respect to lipids was 0.67% (w/w)). Samples were diluted to 20 mg/mL PL concentration with purified NETs in RPMI media and incubated 30 min at 37 °C prior to use. RPMI media alone was used as a control to identify any effect produced by the RPMI media vehicle.

### Captive bubble surfactometer (CBS)

The CBS is a device that allows evaluating the biophysical function of lung surfactant in vitro^69^. This device creates an environment that mimics the situation of a single alveolus in the lung^56,69,70^. A small air-bubble was created inside a chamber filled with buffer (150 mM NaCl, 5 mM Tris (pH 7.4), and 10% (w/v) sucrose). The sample to be tested (150 nL) was applied with a capillary inside the chamber near the surface of the bubble. Adsorption of the material to the air-water interface was thus evaluated during the initial adsorption (IA) period. The shape of the bubble was monitored and recorded for 5 min to observe changes in the surface tension (γ). After adsorption, the chamber was sealed. Compression-expansion cycles of the bubble at 20 cycles/min were then carried out to evaluate the dynamic behavior of the surfactant. These cycles mimic the situation in the alveoli during breathing, while lungs are inflated and deflated during air inspiration and expiration, respectively.

To test the samples from mice, 30 dynamic cycles were carried out at 30 cycles/min, which is the maximum speed allowed by the CBS device, even though mice breathing frequency is higher. Three replicates were performed of each isolated LS, and the average surface activity of the replicates was represented in the graphs.

### Surface balance

To observe and analyze the lateral structure of interfacial surfactant films, surfactant suspensions were spread onto a buffered (150 mM NaCl, 5 mM Tris pH 7.4) sub-phase in a Langmuir surface balance equipped with a continuous Teflon ribbon barrier and a 200 cm^2^ trough (Nima Technology, Coventry). Organic extract from native surfactant was purified from porcine lungs as previously described, and doped with a trace of Alexa488 (Invitrogen) fluorescently-labeled surfactant protein SP-B (F-SP-B, 1% (w/w) with respect to lipids). The sample was dried under nitrogen flow and then under vacuum in an UNIVAP at 37 °C, for 2 h to remove traces of organic solvents. The mixture was reconstituted to 10 mg/mL final PL concentration with buffer 150 mM NaCl, 5 mM Tris pH 7.4. Then, it was labeled with rhodamine-DOPE (Avanti Polar Lipids, Inc.) (0.1% mol of probe/mol of phospholipid each, added in a trace of dimethyl sulfoxide) in the presence or absence of 20 μL of purified NETs in RPMI media with a DNA concentration of 0.65 μg/mL, at 37 °C for 1 h with intermittent shaking (10 min shaking/10 min repose; 1400 rpm in a Thermomixer), taking the surfactant suspension to a final PL concentration of 5 mg/mL. Drops of the surfactant suspension were finally deposited onto the surface of the aqueous sub-phase in the surface balance until a surface pressure of 2 mN/m was achieved. After 10 min to allow the material to stabilize, the interfacial film was compressed at 65 cm^2^/min to a final target pressure of 30 mN/m. After 10 min of further equilibration, Langmuir–Blodgett films were prepared by transferring the interfacial films onto glass cover slides using the COVASP method as described elsewhere^69^ at a transfer rate of 25 cm^2^/min, thus allowing the correct transference without altering the structure of the surfactant layers. Epifluorescence microscopy images were obtained in a Leica DM 4000B microscope, equipped with a camera Hamamatsu Orca-R^2^.

### Western blotting (WB)

Samples for WBs of lung surfactant proteins were prepared by applying 10 μg of surfactant PC per sample. Samples to analyze SP-A and SP-C were loaded under reducing conditions using Laemmli buffer as loading buffer^71^ containing 4% (v/v) β-mercaptoethanol; analysis of SP-B was performed under non-reducing conditions. Samples were boiled at 99 °C, 10 min. They were loaded in 16% (w/v) or 4-20% (w/v) acrylamide gels and later transferred onto a PVDF membrane for 1 h at 300 mA. Membranes were blocked with 5% (w/v) BSA in PBS with 0.05% (v/v) Tween (PBS-T) for 2 h, followed by incubation with primary antibodies overnight. SP-B (1:5000 dilution) or SP-C (1:5000 dilution) antibodies were from Seven Hills Bioreagents^72,73^. SP-A (1:10,000 dilution) antibodies were kindly supplied by Dr. J. Floros from Penn State University, USA^74^. Secondary antibody was anti-rabbit-HRP (Dako) at 1:10,000 dilution for SP-A and 1:5000 dilution for SP-B and SP-C.

To analyze the citH3, 25 μL of each mice BAL-supernatant were mixed with Laemmli buffer with 4%(v/v) β-mercaptoethanol and heated at 99 °C for 10 min. Samples were loaded into 12% (w/v) acrylamide gels and transferred onto nitrocellulose membranes. Membranes were blocked as previously described and incubated with primary anti-citH3 antibody (Abcam ref. #5103) at 1:500 dilution and secondary anti-rabbit antibody (Dako) 1:5000 dilution. In both cases, bands were developed with ECL (Millipore) and densitometry analysis was carried out with ImageJ software.

### Statistics and reproducibility

All statistical analyses were performed on GraphPad Prism 7. For multiple comparisons Two-way ANOVA with Bonferroni post-test and One-way ANOVA with Tukey’s post-test or *t*-test were used as appropriate. All data are presented as mean ± SD. The biological replicates and applied statistical tests are noted in the respective figure legends. Significant differences were considered with a *p*-value of <0.05(*), <0.01(**), <0.001(***), and <0.0001(****).

### Reporting summary

Further information on research design is available in the Nature Research Reporting Summary linked to this article.

## Supplementary information

Supplementary Information

Description of Additional Supplementary Files

Supplementary Data 1

Supplementary Data 2

Reporting Summary

## Data Availability

All the source raw data used for generating main and Supplementary Figures are provided as supplementary excel files.
