# Supplementary material for: SP-D attenuates LPS-induced formation of human neutrophil extracellular traps (NETs), protecting pulmonary surfactant inactivation by NETs
Source: Commun Biol. 2019 Dec 16;2:470. doi: 10.1038/s42003-019-0662-5 (PMC6915734; doi:10.1038/s42003-019-0662-5)
Supplement: Supplementary file 1 — Supplementary Information [file 42003_2019_662_MOESM1_ESM.docx]

**SUPPLEMENTARY FIGURE 1**

**Supplementary Figure 1: Structural analysis of human SP-D from individuals with alveolar proteinosis. *a***, Electrophoretic analysis of hSP-D from PAP. Left, fractions eluted from the purification column were run in an 8% acrylamide gel in the presence of reducing agent (DTT- dithiothreitol); fractions 2-7 contained purified hSP-D. Right, the sample from fraction two (from the left gel) was run in non-reducing (no DTT) and reducing (+DTT) conditions in an 8-15% acrylamide gel; in the absence of DTT the trimer band is observed and in the presence of DTT the main protein band corresponds to the monomer. ***b***, Atomic Force Microscope (AFM) analysis of hSP-D from PAP. Twenty μL of purified hSP-D at 3.4 ng/μL in 150 mM NaCl, Tris 20 mM (pH 7.4) and 5 mM EDTA buffer were incubated for 4-5 minutes onto freshly cleaved mica. The surface was rinsed with 6-7 mL of deionized water to remove buffer components. Finally, the sample was dried under nitrogen air flow. Samples were scanned with an AFM from Nanotec and Point ProbePlus tips, type PPP-NCH (Nanosensors, Neuchâtel, Switzerland).

**SUPPLEMENTARY FIGURE 2**

**Supplementary Figure 2: Linear decrease of NETosis with increased SP-D concentrations.** NETosis kinetics in isolated neutrophils, from healthy human peripheral blood, was assessed by SytoxGreen plate reader assays, where the %DNA released by the cells in the presence of 0.5% (v/v) Triton was considered as 100%. SP-D dose-dependently suppresses LPS (5 μg/mL)-induced NETosis. The regression graph represents the values at final time 240-min, showing a linear decrease of NETosis with increase in the concentration of SP-D (n=3).

**SUPPLEMENTARY FIGURE 3**

**Supplementary Figure 3: SP-D did not show any effect when it was incubated with neutrophils before adding LPS.** NETosis kinetics was assessed by SytoxGreen plate reader assays. Different wells of 96-wells microplate were seeded with 100 μL of a cell suspension containing 50,000 human isolated neutrophils and 5 μM Sytox green. SP-D (at 5, 10 μg/mL) was added to the cells and incubated for 30 minutes at 37 ºC, 5% (v/v) CO_2_ incubator. After incubation, LPS agonist was added at 5 μg/mL and NETosis kinetics was monitored, as previously described. The batch of SP-D used in these experiments was different, for that reason a control with the pre-incubation procedure, explained in the main methods section, was also included (PM-LPS + SP-D10 μg/mL), which showed that this batch of SP-D worked at 10 μg/mL. Calcium concentration of SP-D buffer was adjusted prior to use to 5 mM CaCl_2_. The %DNA released in presence of 0.5% (v/v) Triton was considered as 100%. N=2, error bars represent SD.

**SUPPLEMENTARY FIGURE 4**

**Supplementary Figure 4: SP-D does not affect the PMA- and Ionomycin-induced NETosis**. ***a-c***, NETosis kinetics was assessed by SytoxGreen plate reader assays as described in the methods section. The %DNA released in presence of 0.5% (v/v) Triton was considered as 100%. ***a***, controls to reject any effect of SP-D on neutrophils in the absence of LPS and any effects of SP-D buffer components to reduce LPS-induced NETosis. LPS concentration was 5 µg/mL. ***b*** *and* ***c***, SP-D (at 10, 20 and 40 µg/mL) does not inhibit PMA (25 nM)- or ionomycin (2.5 µM)-induced NETosis.

**SUPPLEMENTARY FIGURE 5**

**Supplementary Figure 5: Low Magnification images taken from figure 2 further confirm that SP-D suppresses LPS-mediated NETosis. *a-b,*** Neutrophils were activated for 2 hours by LPS (***a***) or Ionomycin (***b***) with or without SP-D at two calcium concentrations and imaged after immunostaining for myeloperoxidase (MPO; green) and staining with DAPI for DNA (blue). MPO co-localized to NET-DNA upon stimulating neutrophils with LPS or Ionomycin. ***a***, Pre-incubation of indicated concentrations of SP-D with LPS showed that SP-D suppresses NETosis. ***b***, SP-D did not suppress NETosis induced by ionomycin (n=2, scale bar, 20 μm).

**SUPPLEMENTARY FIGURE 6**

**Supplementary Figure 6: Additional images showing that LPS (0128:B12) induces citrullination of Histone 3 (citH3) during NETosis and that SP-D suppresses LPS-induced citH3 formation.** Neutrophils were activated for 2 hours with buffer control, LPS or ionomycin with or without 20 and 40 μg/mL of SP-D. After fixing, these specimens were immunostained and imaged for citH3 (red) and myeloperoxidase (MPO; green) and staining with DAPI for DNA (blue). LPS and ionomycin, but not buffer control, induced citH3 formation. SP-D suppressed LPS-mediated, but not ionomycin-mediated citH3 formation. ***a***, images were only the immunostaining for citH3 is shown. ***b,*** Images showing the merged channels (citH3-MPO-DAPI); (n=2, scale bar, 40 μm).

**SUPPLEMENTARY FIGURE 7**

**Supplementary Figure 7: Intranasal LPS instillation induces exacerbated CitH3 formation in the airways of SP-D^-/-^ (KO) mice.** Western blot membranes of anti-citH3 for the membranes shown in Figure 4A. WT-PBS (n=6), KO-PBS (n=7), WT-LPS (n=7), KO-LPS (n=7).

**SUPPLEMENTARY FIGURE 8**

**Supplementary Figure 8: Initial adsorption of the LS of WT and KO mice to the air-liquid interface does not differ during LPS-mediated inflammation.** A volume of 150 nL of LS at a PL concentration of 10 mg/mL was applied with a capillary inside the chamber near the surface of the bubble. Adsorption of the material to the air-water interface was evaluated during the Initial adsorption (IA). The shape of the bubble was monitored and recorded during 5 minutes to observe changes in the surface tension (γ). The graph shows the surface tension at the end of the IA for individual mice samples. No differences were observed between the different mouse groups. WT-PBS (n=7), KO-PBS (n=7), WT-LPS (n=9), KO-LPS (n=11); One-way ANOVA with Tukey’s multiple comparison post-test F(3,30)=0.3916, p=0.7599.

**SUPPLEMENTARY FIGURE 9**

**Supplementary figure 9: Surface activity of lung surfactant from WT or KO mice instilled with PBS or LPS.** Surface activity is represented as isotherms obtained from the CBS maintained at 37ºC. One replicate from each individual mouse is represented. For all mice groups, *top row* shows IA to the air-water interface of a bubble over a 5-min period. *Bottom row*, dynamic compression-expansion cycling at 30 cycles/min. PL concentration of LS was 10 mg/mL.

**SUPPLEMENTARY FIGURE 10**

**Supplementary figure 10: Western blots for SP-A, SP-B and SP-C from the different mouse groups.** Western blot membranes for the surfactant proteins SP-A (top), SP-B (middle) and SP-C (bottom) used for densitometry to determine surfactant protein levels. The sample labelled as “control” was always loaded in all WBs to normalize the densitometry results. In case of SP-B and SP-C some samples were repeated in a second run due to technical difficulties in the first gel, as it can be observed in the images. A representative example of these membranes is shown in Figure 7B.

**SUPPLEMENTARY TABLE 1**

**Supplementary Table 1: Parameters defining the behavior of surfactant films during their function inhibition by NETs and restored by the presence of SP-D**

^1^Minimal surface tension after 5 min of IA.

^2^Minimal surface tension after 20 dynamic compression-expansion cycles (at 20 cycles/min).

^3^Maximal surface tension after 20 dynamic compression-expansion cycles (at 20 cycles/min).

^4^ΔA: Increase of area needed to reach minimal surface tension.

All, n= 4 (One-way ANOVA with Tukey’s multiple comparison post-test; IA F(2,10)=5.203, p=0.0283; γ_max_ F(2,10)=18.19, p=0.0005; ΔA F(2,10)=25.91, p=0.0001; *p<0.05, **, p<0.01; ***, p<0.001).
