# Supplementary material for: SP-D attenuates LPS-induced formation of human neutrophil extracellular traps (NETs), protecting pulmonary surfactant inactivation by NETs
Source: Commun Biol. 2019 Dec 16;2:470. doi: 10.1038/s42003-019-0662-5 (PMC6915734; doi:10.1038/s42003-019-0662-5)
Supplement: Supplementary file 2 — Description of Additional Supplementary Files [file 42003_2019_662_MOESM2_ESM.docx]

Supplementary items not contained in the supplementary material PDF:

- Supplementary Data 1: raw data for figures:
  - Figure 1
  - Figure 4
  - Figure 6
  - Figure 7
  - Figure 8
- Supplementary Data 2: raw data for figures:
  - Figure 2
  - Figure 3
  - Figure 4
  - Figure 8
  - Figure 9
