# Supplementary material for: SP-D attenuates LPS-induced formation of human neutrophil extracellular traps (NETs), protecting pulmonary surfactant inactivation by NETs
Source: Commun Biol. 2019 Dec 16;2:470. doi: 10.1038/s42003-019-0662-5 (PMC6915734; doi:10.1038/s42003-019-0662-5)
Supplement: Supplementary file 5 — Reporting Summary [file 42003_2019_662_MOESM5_ESM.pdf]

nature research

Corresponding author(s): DBPR COMMSBIO-18-1107C

Last updated by author(s): 2019/09/10

Reporting Summary

Nature Research wishes to improve the reproducibility of the work that we publish. This form provides structure for consistency and transparency in reporting. For further information on Nature Research policies, see Authors & Referees and the Editorial Policy Checklist.

Statistics

For all statistical analyses, confirm that the following items are present in the figure legend, table legend, main text, or Methods section.

n/a	Confirmed	
☐	☒	The exact sample size (n) for each experimental group/condition, given as a discrete number and unit of measurement
☐	☒	A statement on whether measurements were taken from distinct samples or whether the same sample was measured repeatedly
☐	☒	The statistical test(s) used AND whether they are one- or two-sided<br>*Only common tests should be described solely by name; describe more complex techniques in the Methods section.*
☒	☐	A description of all covariates tested
☐	☒	A description of any assumptions or corrections, such as tests of normality and adjustment for multiple comparisons
☐	☒	A full description of the statistical parameters including central tendency (e.g. means) or other basic estimates (e.g. regression coefficient) AND variation (e.g. standard deviation) or associated estimates of uncertainty (e.g. confidence intervals)
☐	☒	For null hypothesis testing, the test statistic (e.g. F, t, r) with confidence intervals, effect sizes, degrees of freedom and P value noted<br>*Give P values as exact values whenever suitable.*
☒	☐	For Bayesian analysis, information on the choice of priors and Markov chain Monte Carlo settings
☒	☐	For hierarchical and complex designs, identification of the appropriate level for tests and full reporting of outcomes
☒	☐	Estimates of effect sizes (e.g. Cohen's d, Pearson's r), indicating how they were calculated

Our web collection on statistics for biologists contains articles on many of the points above.

Software and code

Policy information about availability of computer code

Data collection	No software was used for data collection
Data analysis	GraphPad Prism version 7

For manuscripts utilizing custom algorithms or software that are central to the research but not yet described in published literature, software must be made available to editors/reviewers. We strongly encourage code deposition in a community repository (e.g. GitHub). See the Nature Research guidelines for submitting code & software for further information.

Data

Policy information about availability of data

All manuscripts must include a data availability statement. This statement should provide the following information, where applicable:

- Accession codes, unique identifiers, or web links for publicly available datasets
- A list of figures that have associated raw data
- A description of any restrictions on data availability

All the source raw data used to produce main and supplementary Figures are provided in the excel files.

Field-specific reporting

Please select the one below that is the best fit for your research. If you are not sure, read the appropriate sections before making your selection.

☒ Life sciences ☐ Behavioural & social sciences ☐ Ecological, evolutionary & environmental sciences

For a reference copy of the document with all sections, see nature.com/documents/nr-reporting-summary-flat.pdf

Life sciences study design

All studies must disclose on these points even when the disclosure is negative.

Sample size	Mice sample size was designed to have a minimum of 6-7 mice per experimental group, to determine the biophysical activity of lung surfactant. Then, the rest of the volume of the collected specimens were used for determining the concentration of surfactant proteins and cholesterol upon availability. In some samples the volume was not enough to determine all the parameters, for that reason we have different sample size in different experimental groups.<br>Immunostained confocal imagining experiments were repeated twice to confirm the results, as it has been done in previous published research many times.<br>The quantification of the DNA release from neutrophils were performed 3 times at different days and with blood obtained from different donors each of the days, as it has been done in previous published research.
Data exclusions	No data exclusion
Replication	The quantification of the DNA release from neutrophils was performed 3-5 times at different days by using different donors<br>Confocal imagining experiments were repeated twice to confirm results.<br>To determine the levels of citH3 in the supernatant of the BAL of samples the samples were run once in the gel. No replication was needed since there was a positive control in all the membranes and multiple individual mouse samples per group used.<br>To determine the levels of DNA and total protein in the supernatant of the BAL the samples were run once in duplicates for each mouse, and the results shown are the average of the two replicates.<br>To determine the levels of cholesterol and surfactant proteins in the lung surfactant the samples were run once upon availability, this LS sample was very limited in volume and it was not possible to test all the individual mice.<br>Biophysical activity of the lung surfactant for each individual mouse was tested in the captive bubble surfactometer, performing 3 replicates for each sample. The same approach was followed with the in-vitro test of organic extract in the presence of NETs or NETs+SP-D.<br>Surface balance experiments were repeated twice.
Randomization	Not applicable
Blinding	Investigators were not blinded. The parameters measured in the study were objective and experimental determinations.

Reporting for specific materials, systems and methods

We require information from authors about some types of materials, experimental systems and methods used in many studies. Here, indicate whether each material, system or method listed is relevant to your study. If you are not sure if a list item applies to your research, read the appropriate section before selecting a response.

Materials & experimental systems

n/a	Involved in the study	
☐	☒	Antibodies
☒	☐	Eukaryotic cell lines
☒	☐	Palaeontology
☐	☒	Animals and other organisms
☐	☒	Human research participants
☒	☐	Clinical data

Methods

n/a	Involved in the study	
☒	☐	ChIP-seq
☒	☐	Flow cytometry
☒	☐	MRI-based neuroimaging

Antibodies

Antibodies used	anti-Surfactant protein D: anti-SP-D were generated in rabbits (Cocalico Biologicals, PA, and USA).<br>anti-Citrullination of Histone 3 (generated in rabbit) purchased from Abcam Ref.#5103<br>anti-Surfactant protein A, generated in rabbits, provided by Dr. Joanna Floros from Penn State University, USA.<br>anti-Surfactant protein B (generated in rabbit) purchased from Seven Hills Bioreagents Ref#WRAB-48604<br>anti-Surfactant protein C (generated in rabbit) purchased from Seven Hills Bioreagents Ref#WRAB-76694
Validation	antiSP-D: Djiadeu, et al., 2017, Apoptosis; Stolley, JM., et al, 2012, Am J Respir Cell Mol Biol.<br>anti-citH3: ab5103 detects a 17 kDa band in single lane Western Blot. Peptide inhibition in Western Blot hasn't been processed. Modification specificity is determined by Peptide Array. ab5103 binds strongly to Histone H3 citrulline 2 + 8 + 17 peptide. Reacts with: Mouse, Rat, Rabbit, Cow, Human, Monkey.<br>Anti-SP-A: Wang, G., et al., 2000, Am J Physiol Lung Cell Mol Physiol<br>Anti-SP-B: reacts with human, mouse, cow & sheep SP-B, other species not tested. Clark JC., et al., 1995, Proc Natl Acad Sci; Gregory, TJ., et al., 1991, J Clin Invest; Nogee, LM., et al., 2000, Am J Respir Crit Care Med.<br>Anti SP-C: reacts with human, mouse mature SP-C, other species not tested. Ross, GF., et al., 1999, Am J Physiol: Lung Cell Mol Physiol.

Animals and other organisms

Policy information about studies involving animals; ARRIVE guidelines recommended for reporting animal research

Laboratory animals	Balb/c wild type (WT) SP-D+/+ and Knock out (KO) SP-D-/- mice were used in this study. Mice were 10-16 weeks old at the time of the experiment.
Wild animals	The study did not involve wild animals
Field-collected samples	The study did not involve field-collected samples.
Ethics oversight	Human and animal study protocols were approved by the Research Ethics Board (REB) of the Hospital for Sick Children, Toronto, Canada.<br>In addition, all animal procedures were approved by the animal facility Toronto Centre for Phenogenomics (TCP) (Toronto, Canada) and performed according to their protocols and ethical guidelines.

Note that full information on the approval of the study protocol must also be provided in the manuscript.

Human research participants

Policy information about studies involving human research participants

Population characteristics	Healthy human subjects that participated as blood donors were males in a range of age 20-40 years old.
Recruitment	Recruited as a blood donor was to be a male over 18 years old.
Ethics oversight	Human study protocols were approved by the Research Ethics Board (REB) of the Hospital for Sick Children, Toronto, Canada.

Note that full information on the approval of the study protocol must also be provided in the manuscript.
